# A methodological review of resilience measurement scales

**DOI:** 10.1186/1477-7525-9-8

**Published:** 2011-02-04

**Authors:** Gill Windle, Kate M Bennett, Jane Noyes

**Affiliations:** 1 Dementia Services Development Centre, Institute of Medical and Social Care Research, Bangor University, Ardudwy, Holyhead Road, Bangor, LL56 2PX Gwynedd, UK; 2 School of Psychology, University of Liverpool, Eleanor Rathbone Building, Bedford Street South, Liverpool, Merseyside L69 7ZA UK; 3 Centre for Health Related Research, Bangor University, Fron Heulog, Ffriddoed Road Bangor Gwynedd LL57 2EF, UK

## Abstract

**Background:**

The evaluation of interventions and policies designed to promote resilience, and research to understand the determinants and associations, require reliable and valid measures to ensure data quality. This paper systematically reviews the psychometric rigour of resilience measurement scales developed for use in general and clinical populations.

**Methods:**

Eight electronic abstract databases and the internet were searched and reference lists of all identified papers were hand searched. The focus was to identify peer reviewed journal articles where resilience was a key focus and/or is assessed. Two authors independently extracted data and performed a quality assessment of the scale psychometric properties.

**Results:**

Nineteen resilience measures were reviewed; four of these were refinements of the original measure. All the measures had some missing information regarding the psychometric properties. Overall, the Connor-Davidson Resilience Scale, the Resilience Scale for Adults and the Brief Resilience Scale received the best psychometric ratings. The conceptual and theoretical adequacy of a number of the scales was questionable.

**Conclusion:**

We found no current 'gold standard' amongst 15 measures of resilience. A number of the scales are in the early stages of development, and all require further validation work. Given increasing interest in resilience from major international funders, key policy makers and practice, researchers are urged to report relevant validation statistics when using the measures.

## Background

International research on resilience has increased substantially over the past two decades [1], following dissatisfaction with 'deficit' models of illness and psychopathology [2]. Resilience is now also receiving increasing interest from policy and practice [3,4] in relation to its potential influence on health, well-being and quality of life and how people respond to the various challenges of the ageing process. Major international funders, such as the Medical Research Council and the Economic and Social Research Council in the UK [5] have identified resilience as an important factor for lifelong health and well-being.

Resilience could be the key to explaining resistance to risk across the lifespan and how people 'bounce back' and deal with various challenges presented from childhood to older age, such as ill-health. Evaluation of interventions and policies designed to promote resilience require reliable and valid measures. However the complexity of defining the construct of resilience has been widely recognised [6-8] which has created considerable challenges when developing an operational definition of resilience.

Different approaches to measuring resilience across studies have lead to inconsistencies relating to the nature of potential risk factors and protective processes, and in estimates of prevalence ([1,6]. Vanderbilt-Adriance and Shaw's review [9] notes that the proportions found to be resilient varied from 25% to 84%. This creates difficulties in comparing prevalence across studies, even if study populations experience similar adversities. This diversity also raises questions about the extent to which resilience researchers are measuring resilience, or an entirely different experience.

One of the main tasks of the Resilience and Healthy Ageing Network, funded by the UK Cross-Council programme for Life Long Health and Wellbeing (of which the authors are members), was to contribute to the debate regarding definition and measurement. As part of the work programme, the Network examined how resilience could best be defined and measured in order to better inform research, policy and practice. An extensive review of the literature and concept analysis of resilience research adopts the following definition. Resilience is the process of negotiating, managing and adapting to significant sources of stress or trauma. Assets and resources within the individual, their life and environment facilitate this capacity for adaptation and 'bouncing back' in the face of adversity. Across the life course, the experience of resilience will vary [10].

This definition, derived from a synthesis of over 270 research articles, provides a useful benchmark for understanding the operationalisation of resilience for measurement. This parallel paper reports a methodological review focussing on the measurement of resilience.

One way of ensuring data quality is to only use resilience measures which have been validated. This requires the measure to undergo a validation procedure, demonstrating that it accurately measures what it aims to do, regardless of who responds (if for all the population), when they respond, and to whom they respond. The validation procedure should establish the range of and reasons for inaccuracies and potential sources of bias. It should also demonstrate that it is well accepted by responders and that items accurately reflect the underlying concepts and theory. Ideally, an independent 'gold standard' should be available when developing the questionnaire [11,12].

Other research has clearly demonstrated the need for reliable and valid measures. For example Marshall et al.[13] found that clinical trials evaluating interventions for people with schizophrenia were almost 40% more likely to report that treatment was effective when they used unpublished scales as opposed to validated measures. Thus there is a strong case for the development, evaluation and utilisation of valid measures.

Although a number of scales have been developed for measuring resilience, they are not widely adopted and no one scale is preferable over the others [14]. Consequently, researchers and clinicians have little robust evidence to inform their choice of a resilience measure and may make an arbitrary and inappropriate selection for the population and context. Methodological reviews aim to identify, compare and critically assess the validity and psychometric properties of conceptually similar scales, and make recommendations about the most appropriate use for a specific population, intervention and outcome. Fundamental to the robustness of a methodological review are the quality criteria used to distinguish the measurement properties of a scale to enable a meaningful comparison [15].

An earlier review of instruments measuring resilience compared the psychometric properties and appropriateness of six scales for the study of resilience in adolescents[16]. Although their search strategy was thorough, their quality assessment criteria were found to have weaknesses. The authors reported the psychometric properties of the measures (e.g. reliability, validity, internal consistency). However they did not use explicit quality assessment criteria to demonstrate what constitutes good measurement properties which in turn would distinguish what an acceptable internal consistency co-efficient might be, or what proportion of the lowest and highest scores might indicate floor or ceiling effects. On that basis, the review fails to identify where any of the scales might lack specific psychometric evidence, as that judgement is left to the reader.

The lack of a robust evaluation framework in the work of Ahern et al. [16] creates difficulties for interpreting overall scores awarded by the authors to each of the measures. Each measure was rated on a scale of one to three according to the psychometric properties presented, with a score of one reflecting a measure that is not acceptable, two indicating that the measure may be acceptable in other populations, but further work is needed with adolescents, and three indicating that the measure is acceptable for the adolescent population on the basis of the psychometric properties. Under this criteria only one measurement scale, the Resilience Scale [17] satisfied this score fully.

Although the Resilience Scale has been applied to younger populations, it was developed using qualitative data from older women. More rigorous approaches to content validity advocate that the target group should be involved with the item selection when measures are being developed[11,15]. Thus applying a more rigorous criterion for content validity could lead to different conclusions.

In order to address known methodological weaknesses in the current evidence informing practice, this paper reports a methodological systematic review of resilience measurement scales, using published quality assessment criteria to evaluate psychometric properties[15]. The comprehensive set of quality criteria was developed for the purpose of evaluating psychometric properties of health status measures and address content validity, internal consistency, criterion validity, construct validity, reproducibility, responsiveness, floor and ceiling effects and interpretability (see Table 1). In addition to strengthening the previous review, it updates it to the current, and by identifying scales that have been applied to all populations (not just adolescents) it contributes an important addition to the current evidence base.

**Table 1:** Scoring criteria for the quality assessment of each resilience measure

	Property	Definition	Quality criteria	
1	Content validity	The extent to which the domain of interest is comprehensively sampled by the items in the questionnaire (the extent to which the measure represents all facets of the construct under question).	**+ 2**	A clear description of measurement aim, target population, concept(s) that are being measured, and the item selection AND target population and (investigators OR experts) were involved in item selection
			
			**? 1**	A clear description of above-mentioned aspects is lacking OR only target population involved OR doubtful design or method
			
			**- 0**	No target population involvement
			
			**0 0**	No information found on target population involvement

2	Internal consistency	The extent to which items in a (sub)scale are intercorrelated, thus measuring the same construct	**+ 2**	Factor analyses performed on adequate sample size (7* #items and > = 100) AND Cronbach's alpha(s) calculated per dimension AND Cronbach's alpha(s) between 0.70 and 0.95
			
			**? 1**	No factor analysis OR doubtful design or method
			
			**- 0**	Cronbach's alpha(s) <0.70 or >0.95, despite adequate design and method
			
			**0 0**	No information found on internal consistency

3	Criterion validity	The extent to which scores on a particular questionnaire relate to a gold standard	**+ 2**	Convincing arguments that gold standard is "gold" AND correlation with gold standard > = 0.70
			
			**? 1**	No convincing arguments that gold standard is "gold" OR doubtful design or method
			
			**- 0**	Correlation with gold standard <0.70, despite adequate design and method
			
			**0 0**	No information found on criterion validity

4	Construct validity	The extent to which scores on a particular questionnaire relate to other measures in a manner that is consistent with theoretically derived hypotheses concerning the concepts that are being measured	**+ 2**	Specific hypotheses were formulated AND at least 75% of the results are in accordance with these hypotheses
			
			**? 1**	Doubtful design or method (e.g.) no hypotheses)
			
			**- 0**	Less than 75% of hypotheses were confirmed, despite adequate design and methods
			
			**0 0**	No information found on construct validity

5	**Reproducibility**			

5.1	Agreement	The extent to which the scores on repeated measures are close to each other (absolute measurement error)	**+ 2**	SDC < MIC OR MIC outside the LOA OR convincing arguments that agreement is acceptable
			
			**? 1**	Doubtful design or method OR (MIC not defined AND no convincing arguments that agreement is acceptable)
			
			**- 0**	MIC < = SDC OR MIC equals or inside LOA despite adequate design and method
			
			**0 0**	No information found on agreement

5.2	Reliability	The extent to which patients can be distinguished from each other, despite measurement errors (relative measurement error)	**+ 2**	ICC or weighted Kappa > = 0.70
			
			**? 1**	Doubtful design or method
			
			**- 0**	ICC or weighted Kappa < 0.70, despite adequate design and method
			
			**0 0**	No information found on reliability

6	Responsiveness	The ability of a questionnaire to detect clinically important changes over time	**+ 2**	SDC or SDC < MIC OR MIC outside the LOA OR RR > 1.96 OR AUC > = 0.70
			
			**? 1**	Doubtful design or method
			
			**- 0**	SDC or SDC > = MIC OR MIC equals or inside LOA OR RR < = 1.96 or AUC <0.70, despite adequate design and methods
			
			**0 0**	No information found on responsiveness

7	Floor and ceiling effects	The number of respondents who achieved the lowest or highest possible score	**+ 2**	=<15% of the respondents achieved the highest or lowest possible scores
			
			**? 1**	Doubtful design or method
			
			**- 0**	>15% of the respondents achieved the highest or lowest possible scores, despite adequate design and methods
			
			**0 0**	No information found on interpretation

8	Interpretability	The degree to which one can assign qualitative meaning to quantitative scores	**+ 2**	Mean and SD scores presented of at least four relevant subgroups of patients and MIC defined
			
			**? 1**	Doubtful design or method OR less than four subgroups OR no MIC defined
			
			**0 0**	No information found on interpretation

In order to calculate a total score + = 2; ? = 1; - = 0; 0 = 0 (scale of 0-18)

SDC - smallest detectable difference (this is the smallest within person change, above measurement error. A positive rating is given when the SDC or the limits of agreement are smaller than the MIC)

MIC - minimal important change \(this is the smallest difference in score in the domain of interest which patients perceive as beneficial and would agree to, in the absence of side effects and excessive cost)s.

SEM -standard error of measurement

AUC - area under the curve

RR - responsiveness ratio

The aims are to:

• Identify resilience measurement scales and their target population

• Assess the psychometric rigour of measures

• Identify research and practice implications

• Ascertain whether a 'gold standard' resilience measure currently exists

## Methods

### Design

We conducted a quantitative methodological review using systematic principles [18] for searching, screening, appraising quality criteria and data extraction and handling.

### Search strategy

The following electronic databases were searched; Social Sciences CSA (ASSIA, Medline, PsycInfo); Web of science (SSCI; SCI AHCI); Greenfile and Cochrane database of systematic reviews. The search strategy was run in the CSA data bases and adapted for the others. The focus was to identify peer reviewed journal articles where resilience was a key focus and/or is assessed. The search strategy was developed so as to encompass other related project research questions in addition to the information required for this paper.

A. (DE = resilien*) and((KW = biol*) or(KW = geog*) or(KW = community))

B. (DE = resilien*) and((KW = Interven*) or(KW = promot*) or(KW = associat*) or(KW = determin*) or(KW = relat*) or(KW = predict*) or(KW = review) or (definition))

C. (DE = resilien*) and ((KW = questionnaire) or (KW = assess*) or (KW = scale) or (KW = instrument))

Table 2 defines the evidence of interest for this methodological review.

**Table 2:** Defining evidence of interest for the methodological review using the SPICE tool.

Setting	Perspective	Intervention	Comparison	Evaluation	Methodological approach
Resilience of people in all age groups, all populations and all settings	Resilience measurement: development, testing or outcome measurement in empirical studies	Scale development and validation studies; quantitative studies that have applied resilience measurement scales. to promote resilience	Controlled intervention studies, before and after studies, intervention studies with no control, validation studies with or without control;	Psychometric evidence and narrative reports of validity assessed against Terwee et al. (2007)	Quantitative

Adapted from Booth [53].

For this review all the included papers were searched to identify, in the first instance, the original psychometric development studies. The search was then further expanded and the instrument scale names were used to search the databases for further studies which used the respective scales. A general search of the internet using the Google search engine was undertaken to identify any other measures, with single search terms 'resilience scale', 'resilience questionnaire', 'resilience assessment', 'resilience instrument.' Reference lists of all identified papers were hand searched. Authors were contacted for further information regarding papers that the team were unable to obtain.

### Inclusion criteria

Peer reviewed journal articles where resilience measurement scales were used; the population of interest is human (not animal research); publications covering the last twenty years (1989 to September 2009). This time-frame was chosen so as to capture research to answer other Resilience and Healthy Ageing project questions, which required the identification of some of the earlier definitive studies of resilience, to address any changes in meaning over time and to be able to provide an accurate count of resilience research as applied to the different populations across the life course. All population age groups were considered for inclusion (children, adolescents/youth, working age adults, older adults).

### Exclusion criteria

Papers were excluded if only the title was available, or the project team were unable to get the full article due to the limited time frame for the review.

Studies that claimed to measure resilience, but did not use a resilience scale were excluded from this paper. Papers not published in English were excluded from review if no translation was readily available.

### Data extraction and quality assessment

All identified abstracts were downloaded into RefWorks and duplicates removed. Abstracts were screened according to the inclusion criteria by one person and checked by a second. On completion full articles that met the inclusion criteria were retrieved and reviewed by one person and checked by a second, again applying the inclusion criteria. The psychometric properties were evaluated using the quality assessment framework, including content validity, internal consistency, criterion validity, construct validity, reproducibility, responsiveness, floor and ceiling effects and interpretability (see table 1). A positive rating (+) was given when the study was adequately designed, executed and analysed, had appropriate sample sizes and results. An intermediate rating (?) was given when there was an inadequate description of the design, inadequate methods or analyses, the sample size was too small or there were methodological shortfalls. A negative rating (-) was given when unsatisfactory results were found despite adequate design, execution, methods analysis and sample size. If no information regarding the relevant criteria was provided the lowest score (0) was awarded.

Study characteristics (the population(s) the instrument was developed for, validated with, and subsequently applied to, the mode of completion) and psychometric data addressing relevant quality criteria were extracted into purposively developed data extraction tables. This was important as a review of quality of life measures indicates that the application to children of adult measures without any modification may not capture the salient aspects of the construct under question [19].

An initial pilot phase was undertaken to assess the rigour of the data extraction and quality assessment framework. Two authors (GW and KB) independently extracted study and psychometric data and scored responses. Discrepancies in scoring were discussed and clarified. JN assessed the utility of the data extraction form to ensure all relevant aspects were covered. At a further meeting of the authors (GW, KB and JN) it was acknowledged that methodologists, researchers and practitioners may require outcomes from the review presented in various accessible ways to best inform their work. For example, methodologists may be most interested in the outcome of the quality assessment framework, whereas researchers and practitioners needing to select the most appropriate measure for clinical use may find helpful an additional overall aggregate score to inform decision making. To accommodate all audiences we have calculated and reported outcomes from the quality assessment framework and an aggregate numerical score (see table 1).

To provide researchers and practitioners with a clear overall score for each measure, a validated scoring system ranging from 0 (low) to 18 (high. This approach to calculating an overall score has been utilised in other research [20] where a score of 2 points is awarded if there is prima facie evidence for each of the psychometric properties being met; 1 point if the criterion is partially met and 0 points if there is no evidence and/or the measure failed to meet the respective criteria. In line with the application of this quality criteria with another methodological assessment [21] a score was awarded under the 'responsiveness' criterion to scales that reported change scores over time.

A number of studies that had used some of the measures provided further data additional to the validation papers, mainly on internal consistency and construct validity. In these cases a score was awarded and an overall score calculated for the relevant criteria. Data regarding the extent to which the measure was theoretically grounded was extracted for critical evaluation by discussion.

## Results

**Figure 1:**
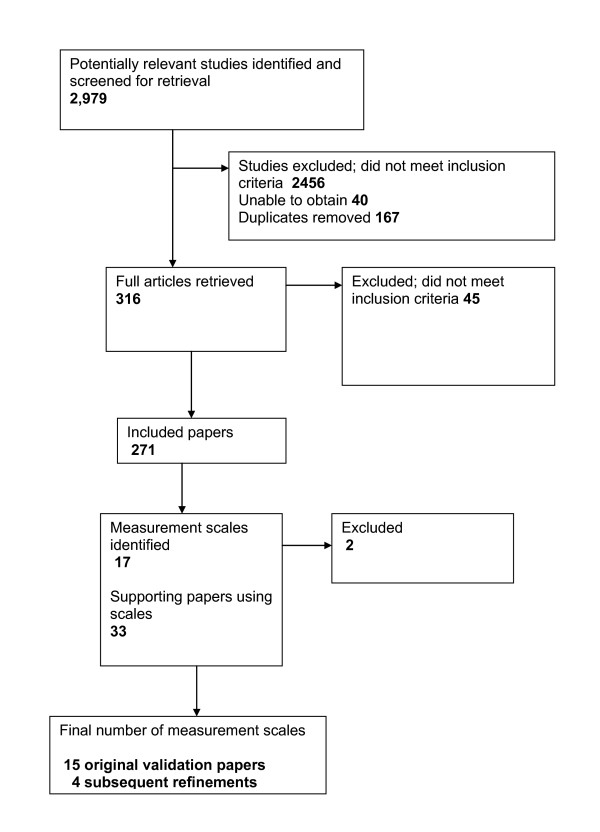
**Flow diagram of review process**.

The search yielded a large amount of potential papers. Figure 1 summarises the process of the review. Seventeen resilience measurement scales were initially identified, and a further 38 papers were identified that had used the scales (see additional file 1). Of these, five papers were unobtainable. One of the measures - the Resiliency Attitudes Scale [22] - was identified through its application in one of the included papers. Although a website exists for the measure, there does not appear to be any published validation work of the original scale development, therefore it was excluded from the final review. Another measure excluded at a later stage after discussion between the authors was the California Child Q-Set (CCQ-Set). Designed to measure ego-resiliency and ego-control, the CCQ-Set does not represent an actual measurement scale, but an assessment derived from 100 observer rated personality characteristics. The final number of measures reviewed was fifteen, with an additional four being reported on that were reductions/refinements of the original measure.

Table 3 provides a description of included measures [14,17,23-42]. In some instances, further development of measures led to reduced or refined versions of the same scale. In these instances results are presented separately for each version of the scale. The mode of completion for all of the measures was self report. The majority (9) focused on assessing resilience at the level of individual characteristics/resources only.

**Table 3:** Description of the Resilience Measures

	Name	**Author(s)**:	Target population	Mode of completion	Number dimensions (items)	Purpose of the measure	**Comments on theory and item selection**:
1a	The Dispositional Resilience Scale (1) (USA/English)	Bartone (1989)	Adults	Self report	3 (45)	Designed to measure psychological hardiness (commitment, control, and challenge). Has been applied to evaluate change over time.	The theoretical background to the development of this scale is derived from the hardiness literature, and in a number of applications it is referred to as a measure of hardiness. As a personality style, it might assist in a resilient response from the individual level, however it is generally regarded as a fixed trait and does not fit well with the notion of resilience as a dynamic process.

1b	The Dispositional Resilience Scale (2) (USA/English)	Bartone (1991)	Adults	Self report	3 (30)	As above	

1c	The Dispositional Resilience Scale (3) (USA/English)	Bartone (1995;2007)	Adults	Self report	3 (15)	As above	

2	The ER 89 (USA/English)	Block & Kremen (1996)	Young adults (18 and 23)	Self report	1 (14)	To measure ego-resiliency (a stable personality characteristic). No clinical applications are suggested.	The construct of Ego Resiliency was first formulated over 50 years ago in the context of personality development. It has a good theoretical basis and has received considerable research attention. It is proposed as an enduring psychological construct that characterizes human adaptability and has been used on occasion by researchers to measure resilience. It is assumed that ego-resilience renders a pre-disposition to resist anxiety and to engage positively with the world. Ego-resiliency does not depend on risk or adversity. It is part of the process of dealing with general, day-to-day change. Ego-resiliency may be one of the protective factors implicated in a resilient outcome, but it would be incorrect to use this measure on its own as an indicator of resilience. Block and Kremen (1996) note that the development of the scale over the years was empirically driven, that 'conceptual decisions were not fully systematic' (p. 352) and changes to the scale have not been recorded properly.

3a	The Connor-Davidson Resilience Scale (CD-RISC) (USA/English)	Connor & Davidson (2003)	Adults (mean age 43.8)	Self report	5 (25)	Developed for clinical practice as a measure of stress coping ability. Five factors (personal competence, trust/tolerance/strengthening effects of stress, acceptance of change and secure relationships, control, spiritual influences). The measure has been used to evaluate change in response to a drug intervention.	The authors take the perspective that resilience is a personal quality that reflects the ability to cope with stress. In their scale development the attempt to identify attributes of resilience is not covered in much depth, and it is not clear why only the work of the three authors cited (Kobasa, Rutter, Lyons) are chosen to identify the characteristics of resilient people. Likewise, the authors make a brief reference to Shackleton's expedition to the arctic, noting that he possessed 'personal characteristics compatible with resilience' (p.77). Research from other authors could potentially have added items to this list. Although this scale was one of the higher scoring ones in the psychometric evaluation and has been applied with an intervention, with reference to our definition, it is an individual level measure that would benefit from more theoretical clarification.

3b	The Connor-Davidson Resilience Scale (CD-RISC) (USA/English)	Cambell-Sills & Stein (2007)	Young adults (mean age = 18.8)	Self report	1 (10)	Short version of 3a. Developed for clinical practice as a measure of stress coping ability.	

4	Youth Resiliency: Assessing Developmental Strengths (YR:ADS) (Canada/English)	Donnon & Hammond (2003, 2007a)	Youth (age 12-17)	Self report	10 (94)	To examine protective factors; intrinsic and extrinsic developmental strengths (family, community, peers, work commitment and learning, school (culture), social sensitivity, cultural sensitivity, self concept, empowerment, self control. Appears to have been developed to generate profiles, and not assess change over time.	The authors summarise the literature with a focus on protective factors and note that youth resiliency is influenced by personal attributes, family characteristics and other external support systems such as peers, the school and the community. In turn, these are described as intrinsic and extrinsic developmental strengths that are related to the development of resilience. The items representing the protective factors were developed from the literature on resilience, protective factors, prevention and child and adolescent development. The dimensions are outlined but the questionnaire is not in the public domain.

5a	The Resilience Scale for Adults (RSA) (Norway/Norwegian	Friborg et al. (2003)	Adults (mean age women = 33.7, men = 36.2)	Self report	5 (37)	To examine intrapersonal and interpersonal protective factors presumed to facilitate adaptation to psychosocial adversities (personal competence, social competence, family coherence, social support, personal structure. The authors note measure can be used in clinical and health psychology as an assessment tool of protective factors important to prevent maladjustment and psychological disorders.	The authors outline evidence from longitudinal research to identify some of the key features of resilient people. These are presented as family support and cohesion, external support systems and dispositional attitudes and behaviours. These were used to define questionnaire items, but it is not clear how the wording for the items was chosen, or whether the target population was involved in item selection. The multi-level nature of the questionnaire is consistent with the assets and resources outlined in our definition.

5b	The Resilience Scale for Adults (RSA)	Friborg et al (2005)	Adults (mean age 22, 24, mid 30s)	Self report	6 (33)	To examine intrapersonal and interpersonal protective factors presumed to facilitate adaptation to psychosocial adversities (personal strength, social competence, structured style, family cohesion, social resources).	As for parent scale.

6	The Resiliency Attitudes and Skills Profile (USA/English)	Hurtes, K. P., & Allen, L. R. (2001).	Youth (age 12-19)	Self report	7 (34)	To measure resiliency attitudes (Insight; independence; creativity; humour; initiative; relationships; values orientation) in youth for recreation and other social services providing interventions.	The authors cite research by some of the key resilience researchers (e.g. Garmezy, Werner, Masten) in the background. Their rationale for their resiliency attitudes is drawn from the qualitative work by Wolin & Wolin (1993) who suggest these characteristics. As this work is drawn from family counseling, the generalisability of the scale is questionable. As with the CD-RISC, other research could potentially inform the dimensions, as the measure is mainly at the level of the individual level, although one of the seven dimensions examines relationships. In terms of measurement construction, the authors specify the procedures they adopted.

7	Adolescent Resilience Scale (Japan/Japanese)	Oshio et al. (2003)	Japanese Youth (19-23 years)	Self report	3 (21)	To measure the psychological characteristics (novelty seeking, emotional regulation, positive future orientation) of resilient Japanese Youth. No clinical applications are reported.	Very little theoretical rationale is presented, and it is unclear as to how the psychological characteristics were chosen to represent resilience.

8	California healthy Kids Survey - The Resilience Scale of the Student Survey (USA/English)	Sun & Stewart (2007)	Primary School Children (mean ages 8.9, 10.05, 12.02)	Self report	12 (34)	To assess student perceptions of their individual characteristics, protective resources from family, peer, school and community (Communication and cooperation, Self-esteem, Empathy, Problem solving, Goals and aspirations, Family connection, School connection, Community connection, Autonomy experience, Pro-social peers, Meaningful participation in community Activity, Peer support). No recommendations by authors regarding to evaluate change.	The introduction in this paper acknowledges resilience as a process. It discusses resilience in relation to Salutogenesis, emphasising the enhancement of protective factors. The authors also discuss resilience within an ecological framework, acknowledging the interactions between the individual, their social environment and the wider community. They acknowledge that resilience encompasses the individual characteristics of the child, family structures and the external environment, and these multiple levels are reflected in the items of the Resilience Scale. The authors also identified peer support at school as an important factor and also added the Peer Support Scale derived from the Perception of Peer Support Scale (Ladd et al., 1996).

9	The Brief Resilience Scale (USA/English)	Smith et al. (2008)	Adults (mean age range 19-62)	Self report	1 (6)	Designed as an outcome measure to assess the ability to bounce back or recover from stress. The authors suggest that assessing the ability to recover of individuals who are ill is important. No clinical applications are reported.	The authors note that most measures of resilience have focused on examining the resources/protective factors that might facilitate a resilient outcome. This scale was developed to have a specific focus on bouncing back from stress. Their arguments are short but clear.. They say that they selected final items from list of potential items but do not identify the full list. The data reduction appears to be based on feedback and piloting of the original list, no empirical validation of the data reduction is reported. In relation to our definition, this scale could be a useful outcome measure in the context of stress.

10	The Child and Youth Resilience Measure (CYRM) (11 countries/11 languages)	Ungar et al. (2008)	Youth at risk (age 12 to 23) in different countries	Self report	4 (28)	To develop a culturally and contextually relevant measure of child and youth resilience across four domains (individual, relational, community and culture). No clinical applications are reported.	The authors do not cite some of the early literature on resilience, but use a definition of their own from previous work to highlight that resilience is a dynamic interplay between the individual and available resources. This interplay involves a process of navigation and negotiation between the individual, their families and the community. They note some of the difficulties in identifying a 'standard' measure of resilience across different cultures and contexts. The project appears to have put a lot of work into the development of the measure, and work was undertaken within 11 countries. The target population was involved in the questionnaire development - at focus groups in 9 countries the youths assisted with the development of the questions which related to the domains defined in previous theoretical work. It appears that the authors have yet to present findings for further application and validation.

11	The Resilience Scale (RS) (Australia/English)	Wagnild & Young (1993)	Adults (some application with 16-23)	Self report	2 (25)	To identify the degree of individual resilience (personal competence and acceptance of self and life); a positive personality characteristic that enhances individual adaptation. sThe measure has had some limited use in evaluating change and has been applied to all age groups from adolescents upwards. Data ranges are suggested which are categorised as low, medium and high.	In the 1993 development paper the authors present a very brief literature review of resilience research. The scale is an individual level measure and was developed from qualitative research with 24 older women who successfully negotiated a major life event. Five themes were derived; equanimity, perseverance, self-reliance, meaningfulness, existential aloneness. The authors state that these were further validated with research literature. However the analytical approach for the five initial components identified in the qualitative work is not outlined, and it is unclear how they came to this conclusion, and then linked it with the research literature. The scale items were derived from verbatim statements from the interviews and from 'generally accepted definitions of resilience'. The definitions are not presented, and it is unclear how comprehensive sampled the items are. The scale was then tested on 39 undergraduate nurses (alpha = 0.89) mean age = 71). This measure appears to have had the widest application out of those identified, and has been used with adolescents, younger and older adults.

12	Psychological Resilience (UK/English)	Windle, Markland & Woods (2008)	Older Adults (subscales previously used with adolescents)	Self report	3 (19)	To assess psychological resilience (self esteem, personal competence and interpersonal control) that acts as a protective factor against risks and adversities. No clinical applications are suggested, although one application examines the moderating effect of psychological resilience on the relationship between ill-health and well-being. The original dimensions have been used to assess change over time.	The measure was developed through secondary data analysis to provide a model of psychological resilience. The literature review in the introduction makes a good case for the respective psychological resources to be considered as indicators of resilience. These are tested and validated empirically. As these items are from established scales with strong underpinning theory that have been applied across populations from adolescents upwards, the measure has the potential to generalise. As yet it has only been used with people aged 50+. In relation our definition, it is an individual level measure.

13	Ego Resiliency (1) (USA/English)	Klohnen (1996)	Adults (18-48)	Self report	4 (20)	To assess the components of ego-resiliency (confident optimism, productive and autonomous activity, interpersonal warmth and insight, skilled expressiveness). No clinical applications are suggested.	The self report measure used in this analysis is based on Block and Block's observer rated assessment of ego resiliency. The author presents a considerable theoretical rationale. The items were drawn from existing data - the California Psychological Inventory (Gough, 1987). This is a 472 item self report inventory with 23 scales that address personality. The full list of items is not presented in the paper and this 29 item measure does not appear to have been utilised in further research. Other comments as for the ER 89.

14	Resilience Scale for Adolescents (READ) (Norway/Norwegian)	Hjemdal et al. (2006a)	Adolescents aged 13-15 years	Self report	5 (39)	To assess the protective resources of personal competence, social competence, structured style, family cohesion and social resources so as to understand stress adaptation.	As with the RSA the authors outline evidence from longitudinal research to identify some of the key features of resilient people. These are presented as family support and cohesion, external support systems and dispositional attitudes and behaviours. The RSA was used as a starting point for the READ items, and were refined based on feedback from seven adolescents. The multi-level nature of the questionnaire is consistent with the assets and resources outlined in our definition.

15	Ego Resiliency (2) (USA/English)	Bromley, Johnson and Cohen (2006)	Adolescents and young adults (mean age = 16 and 22)	Self report	4 (102)	To assess the ego resiliency traits of confident optimism, productive activity, insight and warmth, and skilled expressiveness.	The measure of resilience in this paper was derived from a secondary data set and based on Block and Block's ego resiliency theory. The construct is theoretically established. Items were selected, based on their correspondence with the ER measure of Klohnen (1996) and were drawn from a larger, varied set of personality assessments administered previously. The items included assessments of coping skills, ego-integration, impulse control and responsibility, self esteem, social interaction with peers siblings and adults. It examines resilience at the level of the individual only.

### Overall quality

Table 4 presents the overall quality score of the measures and scores for each quality criteria. With the exception of the Adolescent Resilience Scale and the California Healthy Kids Survey, all of the measures received the highest score for one criteria. Six measures (the RSA, Brief Resilience Scale, Resilience Scale, Psychological Resilience, READ, CD-RISC-10) scored high on two criteria.

**Table 4:** Summary of the quality assessment of the resilience measures

Measure	Content Validity	Internal Consistency	Criterion Validity	Construct Validity	Reproducibility Agreement	Reproducibility reliability (test-retest)*	Responsiveness	Floor/ceiling effect	Interpretability	Total
The Resilience Scale for Adults (RSA - 37 items)	? 1	? 1	0 0	+ 2	0 0	+ 2	0 0	0 0	? 1	7

The Connor-Davidson Resilience Scale (CD-RISC- 25 items)	? 1	? 1	0 0	+ 2	0 0	? 1	? 1	0 0	? 1	7

The Brief Resilience Scale	? 1	+ 2	- 0	+ 2	0 0	? 1	0 0	0 0	? 1	7

The Resilience Scale for Adults (RSA - 33 items)	? 1	+ 2	0 0	+ 2	0 0	+ 2	0 0	0 0	0 0	7

Psychological Resilience	? 1	+ 2	0 0	+ 2	0 0	0 0	0 0	0 0	? 1	6

The Resilience Scale (RS)	+ 2	? 1	0 0	+ 2	0 0	0 0	0 0	0 0	? 1	6

The ER 89	? 1	? 1	0 0	+ 2	0 0	? 1	0 0	0 0	? 1	6

The Connor-Davidson Resilience Scale (CD-RISC - 10 items)	? 1	+ 2	0 0	+ 2	0 0	0 0	- 0	0 0	0 0	5

Resilience Scale for Adolescents (READ)	+ 2	? 1	0 0	+ 2	0 0	0 0	0 0	0 0	0 0	5

The Dispositional Resilience Scale (3)	- 0	+ 2	0 0	? 1	0 0	? 1	0 0	0 0	0 0	4

The Resiliency Attitudes and Skills Profile	+ 2	? 1	0 0	? 1	0 0	0 0	0 0	0 0	0 0	4

Adolescent Resilience Scale	? 1	? 1	0 0	? 1	0 0	0 0	0 0	0 0	? 1	4

Ego Resiliency	? 1	? 1	0 0	+ 2	0 0	0 0	0 0	0 0	0 0	4

The Dispositional Resilience Scale (1)	? 1	? 1	0 0	0 0	0 0	0 0	0 0	0 0	? 1	3

Youth Resiliency: Assessing Developmental Strengths	? 1	+ 2	0 0	- 0	0 0	0 0	0 0	0 0	- 0	3

The Dispositional Resilience Scale (2)	? 1	? 1	0 0	0 0	0 0	0 0	0 0	0 0	? 1	3

The Child and Youth Resilience Measure (CYRM)	+ 2	? 1	0 0	0 0	0 0	0 0	0 0	0 0	0 0	3

California healthy Kids Survey - The Resilience Scale of the Student Survey	0 0	? 1	0 0	0 0	0 0	0 0	0 0	? 1	0 0	2

Ego Resilience (Bromley)	? 1	- 0	0 0	? 1	0 0	0 0	0 0	0 0	0 0	2

### Content validity

Four measures (Resiliency Attitudes and Skills Profile, CYRM; Resilience Scale; READ) achieved the maximum score for content validity and the target population were involved in the item selection. One measure (California Healthy Kids Survey) scored a 0 as the paper did not describe any of the relevant criteria for content validity. The remainder generally specified the target population, had clear aims and concepts but either did not involve the target population in the development nor undertook pilot work.

### Internal consistency

With the exception of Bromley, Johnson and Cohen's examination of Ego Resilience [42], all measures had acceptable Cronbach Alphas reported for the whole scales. The former does not present figures for the whole scale. Alphas were not reported for subscales of the Resilience Scale, the California Healthy Kids Survey, Ego Resiliency and the CD-RISC.

For the Resiliency Attitudes and Skills Profile only one subscale was >0.70. For the RSA, two separate analyses report that one of the six subscales to be <0.70. For the 30 item version of the Dispositional Resilience Scale, the challenge subscale alpha = 0.32, and the author recommends the full scale is used. In the 15 item version, the challenge subscale alpha = 0.70. Bromley et al.'s examination of ego resilience [42] notes that two of the four sub-scales had α < 0.70. One of the five subscales of the READ had α <0.70. Across four different samples the Brief Resilience Scale had alphas >0.70 and <0.95, the YR:ADS, Psychological Resilience and the Adolescent Resilience Scale report α > 0.70 and <0.95 for all subscales, however no factor analysis is reported for the Adolescent Resilience Scale.

### Criterion validity

There is no apparent 'gold standard' available for criterion validity and resilience, and most authors did not provide this information. The Ego Resiliency scale[40] was developed as a self report version of an observer rated version of Ego Resiliency [43] and the latter is stated as the criterion. From two different samples, coefficients of 0.62 and 0.59 are reported. Smith et al. [36] report correlations between the Brief Resilience Scale and the CD-RISC of 0.59 and the ER-89 of 0.51. Bartone [24] reports a correlation of -0.71 between the 30 item Dispositional Resilience Scale and an earlier version of the measure.

### Construct validity

In the absence of a 'gold standard', validity can be established by indirect evidence, such as construct validity [21]. Eight measures achieved the maximum score on this criterion (ER-89, CD-RISC (both 25 and 10 item versions), RSA (37 and 33 item versions), Brief Resilience Scale, RS, Psychological Resilience, the READ and Ego-Resiliency). Evidence for construct validity was lacking in the Dispositional Resilience Scale, YR:ADS, California Healthy Kids Survey and the CYRM.

### Reproducibility - agreement

Information on agreement was not present in any of the papers.

### Reproducibility - reliability (test-retest)

Reliability was investigated for five measures. Three did not specify the type of analysis. The test re-test coefficients are reported for the 15 items Dispositional Resilience Scale, with correlations of 0.78 for commitment, 0.58 for control and 0.81 for challenge. The ER-89 test-retest correlations were 0.67 and 0.51 for two different groups (females and males) however information was lacking about the procedure. For the 37-item RSA the test re-test correlations were >0.70 for all the subscales except the social support (0.69), for the 33 item RSA test-retest correlations were >0.70 for all the subscales. The ICC was 0.87 for the CD-RISC, but the sample size <50 (n = 24) and the type of ICC is not specified. The ICC for agreement for the Brief Resilience Scale was 0.69 in one sample (n = 48) and 0.62 in another (n = 61).

### Responsiveness

Changes over time were examined in the CD-RISC only. Pre and post treatment CD-RISC scores were compared in PTSD treatment responders and non-responders. The patients were receiving drug treatments as part of PTSD clinical trials. No MIC was specified, although they note that response was defined by a score of Clinical Global Improvement with a score of 1 (very much improved); 2 (much improved); 3 or more (minimal or no improvement). It appears that the CD-RISC scores increased significantly with overall clinical improvement, and that this improvement was in proportion to the degree of global clinical improvement. Some limited results are available for the Resilience Scale in Hunter & Chandler [44], who note that post-test scores were significantly higher than pre-test, however the data presented is incomplete and unclear.

### Floor/ceiling effects

The extent of floor or ceiling effects was not reported for any measures.

### Interpretability

For eight measures (RSA 37 items; CD-RISC 25 items; Brief Resilience Scale; Psychological Resilience; The Resilience Scale, the ER-89; the Adolescent Resilience Scale; the Dispositional Resilience Scale), information on sub-groups that were expected to differ was available and in most cases means and standard deviations were presented, although information on what change in scores would be clinically meaningful (MIC) was not specified. Sub group analysis information for the Resilience Scale was available in Lundman, Strandberg, Eisemann, Gustafason and Brulin [45] and Rew, Taylor-Seehafer, Thomas and Yockey [46].

## Discussion

Fifteen measures were identified that propose to measure resilience. All of these measures had some missing information regarding the psychometric properties. Overall, the CD-RISC (25 items), the RSA (37 items) and the Brief Resilience Scale received the highest ratings, although when considering all quality criteria, the quality of these questionnaires might be considered as only moderate. These three aforementioned questionnaires have been developed for use with an adult population.

All but one of the identified resilience scales reflects the availability of assets and resources that facilitate resilience, and as such may be more useful for measuring the process leading to a resilient outcome, or for clinicians and researchers who are interested in ascertaining the presence or absence of these resources. The Brief Resilience Scale states its aim is to assess resilience as an outcome; that is the ability to 'bounce back'. Even so, items in the Brief Resilience Scale, although corresponding with the ability to recover and cope with difficulties, all reflect a sense of personal agency, e.g. 'I usually come through difficult times with little trouble' or 'I have a hard time making it through stressful events'. Most of the measures focus on resilience at the level of the individual only. Two of these (the ER 89 and Psychological Resilience) presented a good theoretical basis to justify the item selection.

Whilst a strong sense of personal agency is important for negotiating adversity, the availability of resources from the level of family and community are also important. The conceptual definition of resilience in the introduction reflects this multi-level perspective of resilience. The development of a measurement instrument capable of assessing a range of protective mechanisms within multiple domains provides an approach to operationalising resilience as a dynamic process of adaptation to adversity [47]. Ideally, measures of resilience should be able to reflect the complexity of the concept and the temporal dimension. Adapting to change is a dynamic process [48]. However only five measures (the CYRM, the RSA, the Resilience Scale of the California Healthy Kids Survey the READ and the YR: ADS) examine resilience across multiple levels, reflecting conceptual adequacy.

### Strengths and weaknesses

To our knowledge, no previous study has systematically addressed the psychometric properties of resilience measures using well-defined criteria. The previous review [16] described a limited number of psychometric properties and did not evaluate them against clear criteria. The improved quality assessment applied in this paper has contributed new evidence to the findings of the previous review. Likewise, extending the inclusion criteria to include all populations, not just adolescents, has increased the number of measures identified and presents more options for a researcher seeking a measure of resilience. However, as yet there is no single measure currently available that we would recommend for studies which run across the lifespan.

Another point relates to the extent to which the measures are culturally appropriate. One scale in particular, the CYRM, received extensive development and piloting within eleven countries, although the authors note that "definitions of resilience are ambiguous when viewed across cultures" (p.174). Thus the meaning of resilience may be culturally and contextually dependent [38].

It is important to identify what the benchmark for 'success' might be for different cultures, who might place different values on such criteria. In terms of the community as a facilitator of resilience, most of the measures for children and adolescents identified in this review have an emphasis on school based resources. This may be appropriate for Western cultures, but be far less so in a country where children do not have automatic access to education. Ungar et al. [38] refer to the 'emic' perspective, which "seeks to understand a concept from within the cultural frame from which the concept emerges" (p.168). From this perspective, the concept of resilience may not necessarily be comparable across cultures. Having said that, Ungar et al [38] found that the key factors underlying resilience were universally accepted across their participating countries, but they were perceived differently by the youths completing the questionnaire. Nevertheless, the setting and circumstances in which a questionnaire is administered play an important role. A good questionnaire seeks to minimise situational effects [12].

As well as reviewing original papers on the psychometric development and validation of resilience measures, this review also sought to identify studies that had used or adapted the respective scales, or contributed to further validation. A further 38 papers were identified, but most studies focussed on the application of scales, and tended to only report information relating to internal consistency. The exceptions here related to four studies that focussed on scale refinement.

The potential limitation of our search strategy should also be considered. As with many reviews, a restriction was placed on the time frame within which to indentify potential studies. If readers wish to be certain no other measures have been developed or new evidence on existing measures published, they should run the search strategy from October 2009 onwards. Likewise, we placed a lower limit of 1989 on the searches, for which the rationale is outlined in the inclusion criteria. We aimed to develop a search strategy sensitive enough to identify relevant articles, and specific enough to exclude unwanted studies. Although we searched 8 databases, we fully acknowledge the issue of potentially missing studies; this is one of the challenges of undertaking a review such as this, Whiting et al.[49] recommend undertaking supplementary methods such as reference screening. We hope that by conducting a general internet search in addition to database searching helps to alleviate the potential for overlooking relevant studies.

It should also be noted that the rating of the measurement scales was hampered by the lack of psychometric information, so it was impossible to give a score on a number of quality criterion, such as reproducibility and responsiveness. We wish to emphasise that this does not necessarily mean that the scale is poor, but would urge researchers to report as much information as possible so as to inform further reviews.

On the other hand, the quality assessment criteria used for this paper could be considered to be overly constraining. However it is one of the few available for evaluating measurement scales, and clearly identifies the strengths and weaknesses of respective measures.

### Recommendations for further research

Our analyses indicate the need for better reporting of scale development and validation, and a requirement for this information to be freely available. Further development and reporting by the authors of the measurement scales could improve the assessments reported here.

Most of the measures advocated application where assessment of change would be required, for example in a clinical setting, or in response to an intervention. An important aspect of three of the criterion (agreement, responsiveness and interpretability) was whether a minimal important change (MIC) was defined. However none of the measures reported a MIC, and it was impossible to receive the maximum score for these criterion. Only one validation paper (the CD-RISC) examined change scores and reported their statistical significance. However it has been noted that statistical significance in change scores does not always correspond to the clinical relevance of effect, which often is due to the influence of sample size [50]. Thus developers of measurement scales should indicate how much change is regarded as clinically meaningful.

As some of the scales are relatively new, and are unlikely as yet to have been adopted into practice, there is scope to improve here. Qualitative research with different patient groups/populations would enable an understanding of how any quantitative changes match with qualitative perspectives of significance. There is also a need for researchers who examine changes scores to present effect sizes, or as a minimum, ensure that data on means, standard deviations and sample sizes are presented. This will enable others who may be considering using a resilience measure in a clinical trial to be able to perform a sample size calculation. However what is lacking from most measures is information on the extent to which measures are responsive to change in relation to an intervention. It is difficult to ascertain whether or not an intervention might be theoretically adequate and able to facilitate change, and whether the measure is able to accurately detect this change.

Also important to note is the absence of a conceptually sound and psychometrically robust measure of resilience for children aged under 12. Only one of the measures, the Resilience Scale of the California Healthy Kids Survey applied this to primary school children (mean ages 8.9, 10.05, 12.02), however this measure scored poorly according to our quality assessment. Resilience research with children has tended to operationalise resilience by looking at ratings of adaptive behaviour by other people, such as teachers, parents, etc. A common strategy is to use task measures which reflect developmental stages [6]. For example Cichetti and Rosgoch [51] examined resilience in abused children and used a composite measure of adaptive functioning to indicate resilience which consists of 7 indicators; different aspects of interpersonal behaviour important for peer relations, indicators of psychopathology and an index of risk for school difficulties.

### Implications for practice

Making recommendations about the use of resilience measures is difficult due to the lack of psychometric information available for our review. As with recommendations in other reviews [21], consideration should be given to the aim of the measurement; in other words, 'what do you want to use it for?' Responsiveness analyses are especially important for evaluating change in response to an intervention [21]. Unfortunately only one measure, the CD-RISC has been used to look at change in response to an intervention. This measure scored also highest on the total quality assessment, but would benefit from further theoretical development.

However five measures (the RSA, the CD-RISC, the Brief Resilience Scale, the ER-89 and the Dispositional Resilience Scale provided test-retest information, and the RSA scored the maximum for this criteria. This provides some indication of the measure's stability, and an early indication of the potential for it to be able to detect clinically important change, as opposed to measurement error. For researchers interested in using another resilience measure to ascertain change, in the first instance we would recommend that reliability (test-rest) for the measure is ascertained prior to inclusion in an evaluation.

None of the adolescent resilience measures scored more than 5 on the quality assessment. The higher scoring RS has been applied to populations across the lifespan from adolescence upwards. However as development was undertaken with older women, it is questionable as to how appropriate this measure is for younger people. Given the limitations, in the first instance, consideration should perhaps be given to measures that achieved the highest score on at least two of the criteria. On that basis the READ may be an appropriate choice for adolescents.

A further important point not covered in the quality assessment criteria related to the applicability of the questionnaire. Questionnaires that require considerable length of time to complete may result in high rates of non-response and missing data. Initial piloting/consultation with qualitative feedback could help identify the questionnaire design that is most likely to be positively received by the target group. As noted above, from a cultural perspective, care needs to be given that the choice of measure is meaningful for the population it is to be applied to. One measure (the CYRM) was developed simultaneously across eleven countries, and may be the best choice for a cross-national survey.

In terms of our findings, for researchers undertaking cross-sectional surveys, especially if undertaking multivariate data analysis, consideration could be given to measures that demonstrate good content and construct validity and good internal consistency. This could provide some assurances that the concept being measured is theoretically robust, that any sub-scales are sufficiently correlated to indicate they are measuring the same construct and that analyses will be able to sufficiently discriminate between and/or soundly predict other variables of interest. The Brief Resilience Scale could be useful for assessing the ability of adults to bounce back from stress, although it does not explain the resources and assets that might be present or missing that could facilitate this outcome. In practice, it is likely that a clinician would need to know an individual's strengths and weaknesses in the availability of assets and resources in order to facilitate interventions to promote development of resilience. Assessing a range of resilience promoting processes would allow key research questions about human adaptation to adversity to be addressed [52]. Identifying protective or vulnerability factors can guide a framework for intervention, for example a preventative focus that aims to develop personal coping skills and resources before specific encounters with real life adversity [47].

## Conclusions

We found no current 'gold standard' amongst 15 measures of resilience. On the whole, the measures developed for adults tended to achieve higher quality assessment scores. Future research needs to focus on reporting further validation work with all the identified measures. A choice of valid resilience measures for use with different populations is urgently needed to underpin commissioning of new research in a public health, human-wellbeing and policy context.

## Competing interests

The lead author is also the developer of one of the scales included in the review (Psychological Resilience). To ensure the fidelity of the review, the measure was reviewed by JN and KB.

## Authors' contributions

GW lead the work-programme of the Resilience Network and was responsible for the search strategy and conceptualisation of the manuscript. She lead the production of the manuscript and reviewed each of the included papers with KB.

KB reviewed the included papers and contributed to the writing of the manuscript.

JN provided methodological oversight and expertise for the review and contributed to the writing of the manuscript.

All authors read and approved the final manuscript.

## Authors' information

Gill Windle PhD is a Research Fellow in Gerontology with expertise in mental health and resilience in later life, and quantitative research methods.

Kate Bennett PhD is a Senior Lecturer in Psychology with expertise in bereavement and widowhood.

Jane Noyes PhD is Professor of Nursing Research with expertise in health services research and evaluation.

## Supplementary Material

Additional file 1 **This file contains references of other papers that used the identified measures**. Click here for file
