# Supplementary material for: A methodological review of resilience measurement scales
Source: Health Qual Life Outcomes. 2011 Feb 4;9:8. doi: 10.1186/1477-7525-9-8 (PMC3042897; doi:10.1186/1477-7525-9-8)
Supplement: Additional file 1 — This file contains references of other papers that used the identified measures. [file 1477-7525-9-8-S1.PDF]

References of include papers that use the reviewed measures (not the primary validation papers – supporting evidence)

Reference:	Measure:
Alexander, D. A., & Klein, S. (2001). Ambulance personnel and critical incidents: Impact of accident and emergency work on mental health and emotional well-being. *British Journal of Psychiatry, 178*, 76-81.	Dispositional resilience scale
Aroian, K. J., & Norris, A. E. (2000). Resilience, stress, and depression among Russian immigrants to Israel. *Western Journal of Nursing Research.Special Issue: Transcultural and Cross-Cultural Nursing Research (the First of Two), 22*(1), 54-67.	The Resilience Scale (RS)
Campbell-Sills, L., Cohan, S. L., & Stein, M. B. (2006). Relationship of resilience to personality, coping, and psychiatric symptoms in young adults. *Behaviour Research and Therapy, 44*(4), 585-599.	Connor Davidson Resilience Scale (CD-RISC)
Christopher, K. A. (2000). Determinants of psychological well-being in I rish immigrants. *Western Journal of Nursing Research, 22*(2), 123-140.	RS
Connor, K. M., Davidson, J. R. T., & Lee, L. C. (2003). Spirituality, resilience, and anger in survivors of violent trauma: A community survey. *Journal of Traumatic Stress, 16*(5), 487-494.	CD-RISC
Connor, K. M., Davidson, J. R. T., & Lee, L. C. (2003b). Spirituality, resilience, and anger in survivors of violent trauma: A community survey. *Journal of Traumatic Stress, 16*(5), 487-494.	CD-RISC
Donnan, T. and Hammond, W. (2007). Understanding the relationship between resiliency and bullying in adolescence. An assessment of youth resiliency in five urban high schools*. Child and Adolescent Psychiatric Clinics of North America, 16*(2), 449-471.	YR:ADSC
Eisenberg, N., Valiente, C., Fabes, R. A., Smith, C. L., Reiser, M., Shepard, S. A., et al. (2003). The relations of effortful control and ego control to children's resiliency and social functioning. *Developmental Psychology, 39*(4), 761-776.	Ego resiliency
Farber, E. W., Schwartz, J. A. J., Schaper, P. E., Moonen, D. J., & McDaniel, J. S. (2000). Resilience factors associated with adaptation to HIV disease. *Psychosomatics: Journal of Consultation Liaison Psychiatry, 41*(2), 140-146.	Dispositional Resilience Scale
Friborg, O., Martinussen, M., & Rosenvinge, J. H. (2006). Likert-based vs. semantic differential-based scorings of positive psychological constructs: A psychometric comparison of two versions of a scale measuring resilience. *Personality and Individual Differences, 40*(5), 873-884.	RSA
Gillespie, B. M., Chaboyer, W., Wallis, M., & Grimbeek, P. (2007). Resilience in the operating room: Developing and testing of a resilience model. *Journal of Advanced Nursing, 59*(4), 427-438.	CS-RISC

Greeff, A. P., & Ritman, I. N. (2005). Individual characteristics associated with resilience in single-parent families. *Psychological Reports, 96*(1), 36-42.	Ego Resiliency
Hart, D., Burock, D., London, B., Atkins, R., & Bonilla-Santiago, G. (2005). The relation of personality types to physiological, behavioural, and cognitive processes. *European Journal of Personality, 19*(5), 391-407.	California Healthy Kids
Hjemdal, O., Friborg, O., Stiles, T. C., Rosenvinge, J. H., & Martinussen, M. (2006). Resilience predicting psychiatric symptoms: A prospective study of protective factors and their role in adjustment to stressful life events. *Clinical Psychology & Psychotherapy, 13*(3), 194-201.	RSA
Kwok, O., Hughes, J. N., & Luo, W. (2007). Role of resilient personality on lower achieving first grade students' current and future achievement. *Journal of School Psychology, 45*(1), 61-82.	Ego resiliency
Lamond, A. J., Depp, C. A., Allison, M., Langer, R., Reichstadt, J., Moore, D. J., et al. (2008). Measurement and predictors of resilience among community-dwelling older women. *Journal of Psychiatric Research, 43*(2), 148-154.	CD_RISC
Letzring, T. D., Block, J., & Funder, D. C. (2005). Ego-control and ego-resiliency: Generalization of self-report scales based on personality descriptions from self, acquaintances, and clinicians. *Journal of Research in Personality, 39,* 395-422*.*	ER-89
Lundman, B., Strandberg, G., Eisemann, M., Gustafson, Y., & Brulin, C. (2007). Psychometric properties of the Swedish version of the resilience scale. *Scandinavian Journal of Caring Sciences, 21*(2), 229-237.	RS
Montross, L. P., Depp, C., Daly, J., Reichstadt, J., Golshan, S., Moore, D., et al. (2006). Correlates of self-rated successful aging among community-dwelling older adults. *American Journal of Geriatric Psychiatry, 14*(1), 43-51.	CD-RISC
Nygren, B., Aléx, L., Jonsén, E., Gustafson, Y., Norberg, A., & Lundman, B. (2005). Resilience, sense of coherence, purpose in life and self-transcendence in relation to perceived physical and mental health among the oldest old. *Aging & Mental Health, 9*(4), 354-362.	RS
Ong, A. D., Bergeman, C. S., Bisconti, T. L., & Wallace, K. A. (2006). Psychological resilience, positive emotions, and successful adaptation to stress in later life. *Journal of Personality and Social Psychology, 91*(4), 730-749.	ER-89
Pinquart, M. (2009). Moderating effects of dispositional resilience on associations between hassles and psychological distress. *Journal of Applied Developmental Psychology, 30*(1), 53-60	RS
Rew, L., Taylor-Seehafer, M., Thomas, N. Y., & Yockey, R. D. (2001). Correlates of resilience in homeless adolescents. *Journal of Nursing Scholarship, 33*(1), 33-40.	RS

Rossi, N. E., Bisconti, T. L., & Bergeman, C. S. (2007). The role of dispositional resilience in regaining life satisfaction after the loss of a spouse. *Death Studies, 31*(10), 863-883.	Dispositional Resilience Scale
Roth, M., & von Collani, G. (2007). A head-to-head comparison of big-five types and traits in the prediction of social attitudes: Further evidence for a five-cluster typology. *Journal of Individual Differences, 28*(3), 138-149.	RS
Smith, B. W., Kay, V. S., Hoyt, T. V., & Bernard, M. L. (2009). Predicting the anticipated emotional and behavioral responses to an avian flu outbreak. *American Journal of Infection Control, 37*(5), 371-380.	Brief Resilience Scale
Sun, J., & Stewart, D. (2007). Development of population-based resilience measures in the primary school setting. *Health Education, 107*(6), 575-599.	California Healthy Kids
Tugade, M. M., & Fredrickson, B. L. (2004). Resilient individuals use positive emotions to bounce back from negative emotional experiences. *Journal of Personality and Social Psychology, 86*(2), 320-333.	ER-89
Ungar, M., Liebenberg, L., Boothroyd, R., Kwong, W. M., Lee, T.Y., Leblanc, J., Duque, L., Maknach, A. (2008). The study of youth resilience across cultures: Lessons from a pilot study of measurement development. *Research in Human Development, 5*(3), 166-180.	CYRM
Wallace, K. A., Bisconti, T. L., & Bergeman, C. S. (2001). The mediational effect of hardiness on social support and optimal outcomes in later life. *Basic and Applied Social Psychology, 23*(4), 267-279.	Dispositional resilience scale
White, B., Driver, S., & Warren, A. (2008). Considering resilience in the rehabilitation of people with traumatic disabilities. *Rehabilitation Psychology, 53*(1), 9-17.	Discusses 2 scales – RSA and CDRS
Windle, G., Woods, B., & Markland, D.A. (2009/in press). Living with ill-health in older age: the role of a resilient personality. *Journal of Happiness Studies.*	Psychological Resilience
Wong, D. F. K. (2008). Differential impacts of stressful life events and social support on the mental health of mainland chinese immigrant and local youth in hong kong: A resilience perspective. *British Journal of Social Work, 38*(2), 236-252.	California Healthy kids
